# Risks of SARS-CoV-2 Breakthrough Infection and Hospitalization in Fully Vaccinated Patients With Multiple Myeloma

**DOI:** 10.1001/jamanetworkopen.2021.37575

**Published:** 2021-11-23

**Authors:** Lindsey Wang, Nathan A. Berger, Rong Xu

**Affiliations:** 1 Center for Artificial Intelligence in Drug Discovery, School of Medicine, Case Western Reserve University, Cleveland, Ohio; 2 Center for Science, Health, and Society, School of Medicine, Case Western Reserve University, Cleveland, Ohio; 3 Case Comprehensive Cancer Center, School of Medicine, Case Western Reserve University, Cleveland, Ohio

## Abstract

This cohort study assesses the risk of breakthrough SARS-CoV-2 infection and hospitalization among fully vaccinated patients with multiple myeloma.

## Introduction

Data from early in the COVID-19 pandemic when vaccines were not available showed that patients with multiple myeloma (MM) were at increased risk for COVID-19 infection and severe outcomes.^1,2^ Recent studies showed a low rate of seroconversion after messenger RNA (mRNA) anti-SARS-CoV-2 vaccination in patients with MM and other hematological malignant neoplasms.^3,4^ However, the risk and outcomes of SARS-CoV-2 breakthrough infection in vaccinated patients with MM remains unknown.

## Methods

This cohort study used the cloud based TriNetX Analytics network platform to access deidentified patient electronic health records (EHRs) from 63 health care organizations in the United States.^5^ The study population comprised 507 288 patients who fulfilled the following inclusion criteria: had recent medical encounter(s) with health care organizations since December 1, 2020; had documented evidence of full vaccination in the EHRs (Pfizer-BioNTech, Moderna, or Johnson & Johnson vaccine) between December 1, 2020, and October 8, 2021; and had no prior COVID-19 infection. EHR data are deidentified, and this study was exempt from institutional review board approval and informed consent per the US Federal Policy for the Protection of Human Subjects. We tested whether fully vaccinated patients with MM had higher risk for breakthrough infections than individuals without cancer after propensity score matching for demographics, adverse socioeconomic determinants of health, transplant procedures, comorbidities, vaccine types, and medications. Kaplan-Meier analysis was used to estimate probability of breakthrough infections starting 14 days after full vaccination. Comparisons between cohorts were made using Cox proportional hazards model and hazard ratio (HR). We tested whether hospitalization rates differed between patients with MM with breakthrough infections and propensity score–matched patients with MM without breakthrough infections. Statistical tests were either conducted within the TriNetX Analytics Platform or using R statistical software (version 3.6.3) with significance set at *P* < .05 (2-sided). Details of TriNetX, study population, and statistical analysis were described in the eMethods in the Supplement. This study followed the Strengthening the Reporting of Observational Studies in Epidemiology (STROBE) reporting guidelines.^6^

## Results

The characteristics of the fully vaccinated population with MM and the population of patients without cancer are shown in the Table. Among 1182 vaccinated patients with MM, 33.8% had monoclonal gammopathy of undetermined significance (MGUS), 11.7% were in relapse, 88.7% had never achieved remission, 60.0% had chemotherapy, 50.3% had targeted therapy, 12.1% had radiation therapy, and 26.5% had stem cell transplant; mean (SD) blood lymphocyte count was 2.08  × 10^9^/L (12.2 × 10^9^/L). Among 187 patients with MM with SARS-CoV-2 breakthrough infections, 34.8% had MGUS, 15.5% were in relapse, 86.6% had never achieved remission, 64.2% had chemotherapy, 54.3% had targeted therapy, 11.2% had radiation therapy, and 27.8% had stem cell transplant; mean (SD) blood lymphocytes count was 1.63 × 10^9^/L (2.01 × 10^9^/L). The overall risk of SARS-CoV-2 breakthrough infections was 15.4% in the MM population and 3.9% in the noncancer population. After propensity score matching for demographics, adverse socioeconomic determinants of health, transplant procedures, comorbidities, vaccine types, and medications, patients with MM remained at significantly increased risk for breakthrough infections compared with matched patients without cancer (HR, 1.34; 95% CI, 1.06-1.69). The estimated probability of hospitalization at the end of the time window (October 8, 2021) was 34.4% for patients with MM with breakthroughs, compared with 4.5% for matched patients without breakthroughs (HR, 15.9; 95% CI, 6.2-40.3) (Figure).

**Table.** Characteristics of the Vaccinated Populations With MM and Without Cancer (as of October 8, 2021) in the TriNetX Database^a^

Patient characteristics (fully vaccinated)	Multiple myeloma	Without cancer	*P* value
Total No. of patients	1182	506 106	
Age, mean (SD), y	68.0 (11.6)	51.3 (20.8)	<.001
Gender			
Female	48.5	55.5	.001
Male	51.5	44.4	
Race and ethnicity, %			
White	61.4	60.4	.47
Black or African American	27.5	14.9	<.001
Hispanic or Latino	6.2	13.4	<.001
Asian	3.9	9.1	<.001
Unknown	6.8	14.6	<.001
Comorbidity, %			
Hypertension	60.2	17.8	<.001
Heart diseases	22.5	3.9	<.001
Type 2 diabetes	22.2	6.5	<.001
Overweight or obesity	19.1	7.7	<.001
Cerebrovascular diseases	13.8	2.7	<.001
Chronic lung diseases	21.6	6.5	<.001
Chronic kidney disease	26.2	2.4	<.001
Liver diseases	16.2	1.9	<.001
HIV	1.3	0.3	<.001
Alcohol use	2.2	0.8	<.001
Tobacco use	8.5	2.3	<.001
Substance use disorders	12.6	3.2	<.001
Organ or tissue transplant, %	25.8	0.4	<.001
Adverse socioeconomic determinants of health, %	2.8	1.1	<.001
Full vaccination type, %			
Pfizer-BioNTech	77.0	90.1	<.001
Moderna	22.2	8.5	<.001
Johnson & Johnson	0.8	1.4	.12
COVID-related medications, %			
Dexamethasone	62.1	9.3	<.001
Hydrocortisone	28.2	4.5	<.001
Ibuprofen	25.6	11.8	<.001
Methylprednisolone	40.1	13.6	<.001
Prednisone	35.6	8.3	<.001
Naproxen	13.5	5.4	<.001
Remdesivir	0	0.002	.87
Tocilizumab	0.9	0.06	<.001
Lopinavir	0.9	0.014	<.001
Ritonavir	0.9	0.07	<.001
Imdevimab	0	0.027	.57

^a^ Demonstrated risk factors for COVID-19 infections or severe outcomes that occurred any time before to the same day as index event of full vaccination are shown. The status of adverse socioeconomic determinants of health was based on the *International Statistical Classification of Diseases and Related Health Problems, Tenth Revision (ICD-10)* code “Persons with potential health hazards related to socioeconomic and psychosocial circumstances” (Z55-Z65), which includes “Problems related to education and literacy” (Z55), “Problems related to employment and unemployment” (Z56), “Occupational exposure to risk factors” (Z57), “Problems related to housing and economic circumstances” (Z59), “Problems related to upbringing” (Z62), among others.

**Figure.**
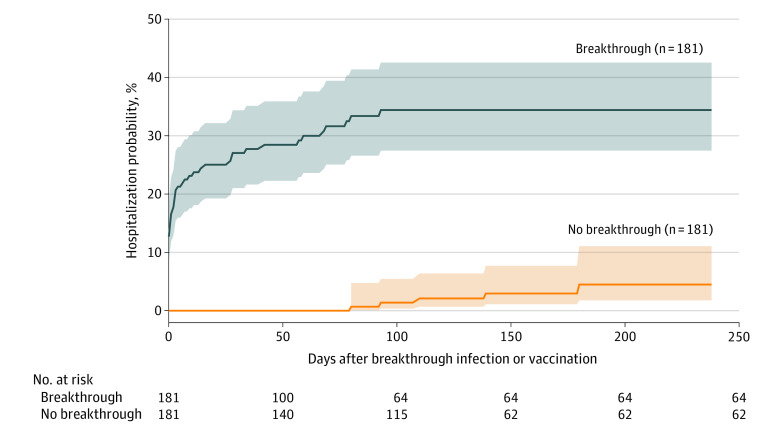
Risk of Hospitalization for Patients With vs Without Breakthrough COVID-19 Infection Kaplan-Meier curves for hospitalization in the breakthrough cohort (fully vaccinated patients with breakthrough infections) with hospitalizations followed starting from the day of breakthrough infections up to October 8, 2021, and in the no-breakthrough cohort (fully vaccinated patients without breakthrough infections) with hospitalizations followed starting at 14 days after full vaccinations up to October 8, 2021. The 2 cohorts were propensity score matched for demographics, adverse socioeconomic determinants of health, transplants, comorbidities, characteristics of multiple myeloma (status, stage, lymphocyte counts), COVID-19-related medications, multiple myeloma treatments (chemotherapy, target therapy, radiation therapy, and stem cell transplant), and vaccine types. Shaded areas represent 95% CIs.

## Discussion

This study found that patients with MM were at increased risk of breakthrough infections and that breakthrough infections were associated with increased risk for hospitalization. These findings raise consideration for the development and implementation of enhanced mitigation strategies and the need for studies to evaluate the timing and impact of vaccine boosters in this unique, immunosuppressed population. Despite the limitations inherent to observational analysis based on patient EHRs, our study leveraged a federated nationwide and real-time patient EHR database that allowed us to electronically monitor the risks and outcomes of vaccine breakthrough infections in a real-world vulnerable population (ie, patients with MM).

## Notes

Supplement. **eMethods. **Additional Study Methods
