# Supplementary material for: Risks of SARS-CoV-2 Breakthrough Infection and Hospitalization in Fully Vaccinated Patients With Multiple Myeloma
Source: JAMA Netw Open. 2021 Nov 23;4(11):e2137575. doi: 10.1001/jamanetworkopen.2021.37575 (PMC8611483; doi:10.1001/jamanetworkopen.2021.37575)
Supplement: Supplement. — eMethods. Additional Study Methods [file jamanetwopen-e2137575-s001.pdf]

Supplemental Online Content

Wang L, Berger NA, Xu R. Risks of SARS-CoV-2 breakthrough infection and hospitalization in fully vaccinated patients with multiple myeloma. *JAMA Netw Open*. 2021;4(11):e2137575. doi:10.1001/jamanetworkopen.2021.37575

eMethods. Additional Study Methods

This supplemental material has been provided by the authors to give readers additional information about their work.

© 2021 Wang L et al. *JAMA Network Open.*

eMethods. Additional Study Methods

Database description and Study population

This study used the cloud based TriNetX Analytics network platform that allows access to de-identified patient electronic health records from 63 health care organizations in the United States. The study population comprised 473,244 patients fulfilled the following inclusion criteria: had recent medical encounter(s) with healthcare organizations since December 1, 2020, had documented evidence of full vaccination in the EHRs (received both doses of Pfizer-BioNTech or Moderna mRNA vaccine, or single dose of Johnson & Johnson (J&J) vaccine) between December 1, 2020 and October 8, 2021, and had not contracted COVID-19 prior to vaccination. The study population included 1,154 MM and 472,090 non-cancer patients (as the comparison group).

TriNetX Analytics is a federated cloud-based network providing web-based real-time secure access to patient EHRs from hospitals, primary care and specialty treatment providers of diverse geographic locations, age groups, race/ethnic groups, and income levels. Though the EHR data are de-identified, end-users can use web-based TriNetX Analytics built-in functions (e.g., propensity score matching, risk, time trend analysis, Kaplan-Meier survival) that work on underlying patient-level EHR data for cohort selection and matching, analyzing incidence and prevalence of events in a cohort, comparing characteristics and outcomes between matched cohorts, among others. Multiple studies have used TriNetX to study risk, disparity, sequelae, temporal trends, clinical characteristics, and outcomes of COVID-19.

© 2021 Wang L et al. *JAMA Network Open.*

The status of COVID-19 was based on ICD diagnosis code of "COVID-19" (U07.1) or lab-test confirmed presence of "Sars coronavirus 2 and related RNA" (TNX:LAB:9088). The status of full vaccination was based on the CPT code "Immunization administration by intramuscular injection of severe acute respiratory syndrome coronavirus 2 (SARS-CoV-2) (Coronavirus disease [COVID-19]) vaccine, mRNA-LNP, spike protein, preservative free, 30 mcg/0.3mL dosage, diluent reconstituted; second dose" (0002A) by Pfizer-BioNTech, "Immunization administration by intramuscular injection of severe acute respiratory syndrome coronavirus 2 (SARS-CoV-2) (Coronavirus disease [COVID-19]) vaccine, mRNA-LNP, spike protein, preservative free, 100 mcg/0.5mL dosage; second dose" (0012A) by Moderna and "Immunization administration by intramuscular injection of severe acute respiratory syndrome coronavirus 2 (SARS-CoV-2) (coronavirus disease [COVID-19]) vaccine, DNA, spike protein, adenovirus type 26 (Ad26) vector, preservative free, 5x10^10 viral particles/0.5mL dosage, single dose" (0031A) by J.&J.

The status of MM was based on the ICD-10 diagnosis code of "Multiple Myeloma" (C90.0), and cancer on the ICD-10 diagnosis code of "Neoplasms" (C00–D49). For breakthrough outcome measures, the status of hospitalization was based on CPT code "Hospital Inpatient Services" (013659).

Statistical analysis

All the statistical analyses were performed within TriNetX Analytics Platform using its built-in functions unless it was specified otherwise.

© 2021 Wang L et al. *JAMA Network Open.*

(1) We tested whether fully vaccinated MM patients had higher risk for breakthrough infections than matched non-cancer patients. MM cohort and non-cancer cohort were propensity-score matched for (a) demographics (age, gender, race/ethnicity), (b) adverse socioeconomic determinants of health (SDOHs) related to education, unemployment, occupational hazard exposure, housing and economic circumstances, among others, (c) transplant procedures, (d) comorbidities including hypertension, heart disease, cerebrovascular diseases, obesity, type 2 diabetes, chronic lung diseases, liver diseases, chronic kidney diseases, smoking, alcohol drinking, substance use disorders that are known risk factors for COVID-19 infection or severe outcomes from COVID-19 infection [33-39], and (e) vaccine types (Pfizer, Moderna, J&J). TriNetX built-in propensity-score matching function was used (1:1 matching using a nearest neighbor greedy matching algorithm with a caliper of 0.25 times the standard deviation) for controlling for the list of variates described above. The outcome was COVID-19 infections followed starting 14 days after patients received the second dose of Pfizer-BioNTech or Moderna vaccine or single dose J.&J. vaccine up to the end of time window (October 8, 2021). Kaplan-Meier analysis was used to estimate the probability of breakthrough infections from 14 days after full vaccination up to October 8, 2021. Comparisons between cohorts were made using a log-rank test and the hazard ratio (HR) was used to describe the relative risk of breakthrough infections based on comparison of time to event rates. The proportional hazard assumption was tested using the generalized Schoenfeld approach.

(2) We tested whether fully vaccinated patients with breakthrough infections had different risk for hospitalization compared with a matched cohort without

© 2021 Wang L et al. *JAMA Network Open.*

breakthrough infections. Cohorts were propensity-score matched for demographics, SDOHs, transplants, comorbidities and vaccine types. For the breakthrough cohort, Kaplan-Meier analysis was used to estimate the probability of hospitalization starting from the day of infection up to October 8, 2021. For the non-breakthrough cohort, Kaplan-Meier analysis was done to estimate the probability of hospitalization starting from 14 day after vaccination up to October 8, 2021. Hazard ratios were used to compare outcomes in matched cohorts. Separate analysis was performed for MM and non-cancer patients.

Statistical tests were conducted with significance set at P value < 0.05 (two sided) using R, version 3.6.3.

© 2021 Wang L et al. *JAMA Network Open.*
